# Radical Prostatectomy: An Option for High-Risk Prostate Cancer

**DOI:** 10.1155/2012/410246

**Published:** 2011-10-11

**Authors:** S. Rausch, C. Schmitt, T. Kälble

**Affiliations:** Department of Urology, Fulda Civic Hospital, Pacelliallee 4, 36043 Fulda, Germany

## Abstract

*Introduction*. High-risk prostate cancer represents a therapeutic challenge. The role of radical prostatectomy (RP) in patients with extreme PSA values is under discussion. *Material and Methods*. We retrospectively analysed our data of 56 consecutive patients with preoperative PSA ≥ 40 mg/mL undergoing open radical retropubic prostatectomy from 1999 to 2009. Patient survival and time to PSA recurrence were recorded, and the Kaplan-Meier survival analysis was performed. Postoperative quality of life and functional status were investigated using a SF-12 questionnaire and determining the number of pads used per day. *Results*. Overall 56 patients were available for followup after a median time of 83.84 months. Locally advanced carcinoma was present in 84% while 16% of patients had organ-confined stages. A positive nodal status was observed in 46%. Overall survival was 95% at five and 81% at 10 years. Cancer-specific survival was 100% for five years and 83% for 10 years. Corresponding biochemical recurrence-free survival was low (52% and 11%, resp.). Quality of life and functional outcomes were favourable. *Conclusions*. In patients with PSA ≥ 40 mg/mL, RP allows long-term control, exact planning of adjuvant treatment, and identification of curable disease.

## 1. Introduction

Prostate cancer is an important medical issue with a high complexity regarding stage classification and risk-adapted multidisciplinary treatment. As the consensus towards the therapy of localised prostate cancer is broad, radical prostatectomy (RP) is an established surgical approach based on reliable clinical data. High-risk prostate cancer is defined as PSA > 20 ng/mL, Gleason 8–10 or clinical stage ≥ T2c. RP is also considered as first-line treatment for higher-risk strata whereas the scientific evidence for patient outcomes, especially those with elevated PSA values greater than 50 ng/mL, is comparably low [1]. Based on our own single centre experience and the implementation of available published data, our investigation targets this relevant clinical topic.

## 2. Material and Methods

We retrospectively analysed our data of 56 consecutive patients with an elevated PSA ≥ 40 ng/mL who underwent radical retropubic prostatectomy with iliac lymphadenectomy from 1999 to 2009 with followup to October 2010. The template of LAD consisted in external, internal iliac, and obturatorial lymph nodes. A nerve-sparing procedure was not conducted. We examined patient survival, time to PSA recurrence, and cancer-related survival. Complete followup was available for 54/56 patients (96%). A Kaplan-Meier analysis was performed to analyse overall (OS) and cancer-specific survival (CSS) and time to biochemical recurrence (BCR), which was defined as postoperative PSA ≥ 0.4 ng/mL or PSA rise while receiving androgen deferral treatment. To assess postoperative quality of life, a SF-12 questionnaire was used. For the evaluation of postoperative continence, the number of used pads per day was interrogated using a patient questionnaire.

## 3. Results

In our cohort, 56 consecutive patients with a preoperative PSA ≥ 40 ng/mL (median: 54.2 ng/mL) were available for the analysis. Mean age at surgery was 66.81 years. Median followup was 83.3 months (IQR: 37.57 to 109.43). Patient characteristics, the distribution of preoperative staging, biopsy Gleason's score, and clinical stages are demonstrated in Table 1.

With regard to pathological staging, a predominance of locally advanced cancer is characterized by 84% stage pT3/pT4 prostate carcinoma whereas, in 16% of patients, the prostate carcinoma was organ confined. A positive nodal status was observed in 46% of patients in our study collective. Pathologic Gleason's scores of ≤6 were observed in 30% of cases, the incidence of Gleason's scores ≥8 was 24%. Surgical margins were positive in 26 patients (46%). The majority of patients (68%) underwent additional hormonal treatment, which was applied according to institutional protocols. From 1999 to 2004, 17 patients (30%) received neoadjuvant hormone ablation with LH-RH analoga. Adjuvant hormonal therapy was applied to 38% either by orchidectomy or postoperative pharmacological (LH-RH agonist) androgen withdrawal. Half of the patients received adjuvant radiation therapy. Postoperative pathological staging, grading, and adjuvant treatment measures are summarized in Table 2. 

**Figure 1:**
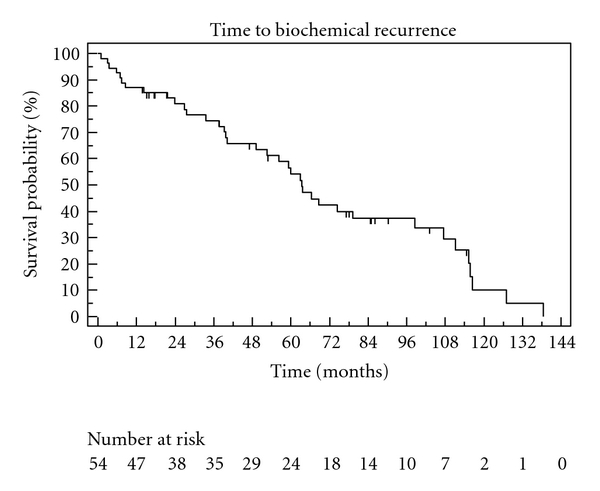
Biochemical recurrence.

**Figure 2:**
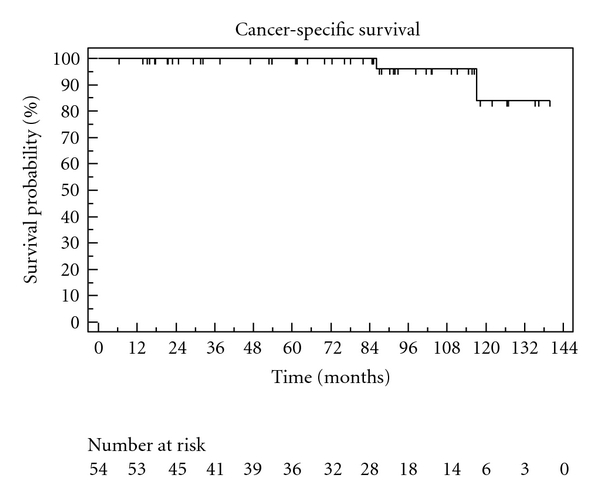
Cancer-specific survival.

**Figure 3:**
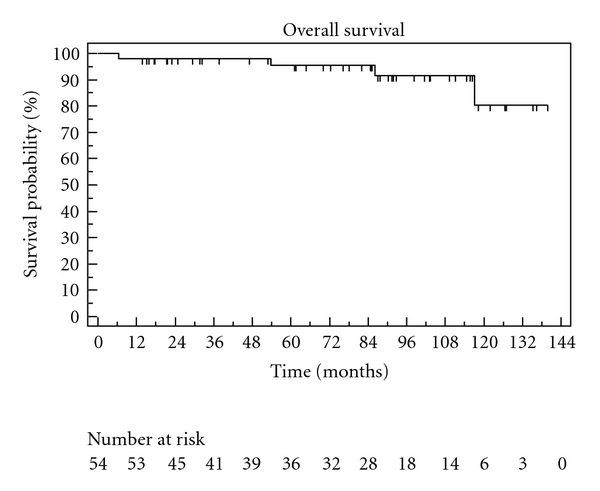
Overall survival.

The Kaplan-Meier curves describing overall survival, CSS, and biochemical recurrence are shown in Figures 1, 2, and 3. Overall survivals at five and ten years were 95% and 81%. The biochemical recurrence-free survival was 52% at five and 11% at ten years. Cancer-specific survival varied only marginally from OS (100% in five years, 83% in ten years), as only two of four deaths in our cohort were caused by prostate cancer. Postoperative continence and quality-of-life results were available through 70% of returned questionnaires in our study. Regarding postoperative quality of life, 75% of the patients described their general state of health as good or very good (SF-12). At last followup, 88% in our study at used maximum one pad per day (Table 2).

## 4. Discussion

In the PSA era, the proportion of men treated with RP for high-risk prostate cancer has decreased while in contrast the number of patients undergoing surgery for low-risk cancer is increasing. Nevertheless, the currently used risk groups remain predictive of patient outcomes [1]. The pretreatment risk stratification for patients diagnosed with high-risk prostate cancer is commonly based on the classification system of D'Amico et al. which includes PSA value (>20 ng/mL), biopsy Gleason's score (8–10), and clinical T-stage (cT2c or more) [2]. Based on the same data, for radical prostatectomy risk, stratification is available not only for biochemical recurrence but also for disease progression and survival [3]. This observation is crucial as the impact of biochemical recurrence does not automatically influence clinical progression and overall survival. To improve prostate cancer risk assessment, Cooperberg et al. established a scoring system based on the CaPSURE database which allows the prediction of clinically more relevant endpoints such as development of metastases, cancer-specific mortality, and overall survival [4]. In our cohort, we reviewed patients with a PSA threshold of >40 ng/mL in order to assess the outcome of individuals with an elevated risk profile.

In a retrospective, multi-institutional analysis of 712 patients, Spahn et al. assessed additional high-risk factors for individuals with pretreatment PSA > 20 ng/mL undergoing RP. Biopsy Gleason's score ≥8 was identified as a strong predictor of progression and survival leading to a cancer-specific mortality of 35% in 10 years, whereas biopsy Gleason's scores smaller or equal to 7 lead to a low cancer-specific mortality of 5% [5]. In their review, Karnes et al. specify the outcome of 1513 men from the Mayo Clinic cohort that were classified into the high-risk group according to the D'Amico criteria. Median followup was 7.7 years, and survival analysis revealed a ten year overall survival of 80% (95% cancer specific). Also, 55% of patients were free of biochemical recurrence in ten years, 90% showed no local recurrence, and 89% no systemic progression [1]. 

In our study, we observed comparable long-term results of 95% overall survival at five years and 81% at 10 years. Only two patients (4%) died from prostate cancer. The biochemical recurrence-free rate at five years was 52%, however, only 11% at 10 years. Apart from the advantage of local disease control, 16% of our patients showed an organ-confined potentially curable tumour stage. Inman et al. analysed the Mayo Clinic data with PSA values between 50 and 100 ng/mL after radical prostatectomy and observed a lower biochemical relapse rate of 40% at 10 years [6]. Another study group observed PSA failures of 27% at five years for patients with elevated PSA of 50 to 100 ng/mL [7]. In a retrospective analysis, Gontero et al. found 48 patients with a PSA ≥ 100 ng/mL treated with RP. In this subset of patients, 8.3% could be cured by surgery alone at a median followup of 78.8 months. Ten-year cancer-specific survival accounted for 79.9%, however, significantly decreased in comparison to lower PSA thresholds in their study [8]. Meng et al. observed that, in the USA, patients with high-risk prostate cancer are significantly less likely to be treated by RP than by primary hormonal or radiation therapy [9]. Nevertheless, the aforementioned cancer-specific and overall survival rates indicate that high-risk prostate cancer patients stand to benefit from radical prostatectomy. The variety of biochemical outcomes in the literature may be the result of different application of adjuvant and salvage therapies in the respective cohorts. In our study, adjuvant and neoadjuvant treatment was applied to 38% and 30% of patients. Fifty percent underwent adjuvant radiation therapy. Walz et al. investigated the pathological characteristics and rates of biochemical recurrences after RP in men with advanced prostate cancer according to the D'Amico classification. The authors observed favourable pathology (organ-confined, negative surgical margins, Gleason's score ≤7) in 13.7% of clinical T3 carcinoma, 16.4% of patients with a biopsy Gleason's score ≥8, and 21.4% for the D'Amico high-risk group. Patients with an elevated PSA ≥ 20 mg/mL showed a favourable pathology in 21.6% of cases. The presence of more than one risk factor led to a decrease in biochemical recurrence-free survival [10]. In the authors's opinion, it is questionable, whether a PSA of >20 or >40 is able to discern a high-risk PCA group with regard to operative treatment and outcome although biochemical relapse rates occur to grow with high preoperative levels of PSA. 

From our own observation and in line with the Mayo Clinic's analysis with about 60% of high-risk stratified patients presenting with organ-confined stages, allowing long-term local disease control in 90% of all patients, there is no rationale to deprive patients of radical surgery [1]. The fact that primary RP goes in line with fundamental pathologic information offers the possibility to apply adjuvant treatment to selected patients and to avoid hormonal or radiation overtreatment. Immediate androgen deprivation after radical treatment has shown to improve patient survival in locally advanced stages [11], and adjuvant androgen withdrawal has proven to be beneficial for patients with positive nodal status [12] while adjuvant radiotherapy can preserve local control in extraprostatic growth and positive surgical margins [13]. In a Cochrane database review, Kumar et al. found no significant improvement on overall survival by neoadjuvant hormonal treatment prior to RP [14]. As the patient cohort in our investigation is recruited from 1999 to 2009, in an early subset of patients (17) from 1999 to 2004, neoadjuvant hormonal treatment has been performed. 

Important issues regarding the surgical treatment of high-risk prostate cancer are operative feasibility, quality of life, and functional outcome. In a single centre, single surgeon study of 288 men treated with radical prostatectomy in a high-risk setting (defined as PSA ≥ 15 ng/mL, ≥cT2b or Gleason's score 8 to 10), Loeb et al. observed a potency rate of 62% and a continence rate of 92% within 10 years [15]. Gontero et al. compared a series of patients with clinically advanced prostate cancer undergoing RP to a control group of clinically organ-confined disease and found no significant differences in surgical morbidity apart from transfusion rate, operation time, and lymphoceles [16]. In our investigation, 75% of patients describe themselves in a good or very good status of health (SF-12) at followup. Favourable functional outcome is documented by a rate of 88% of patients requiring at maximum one pad per day. Nevertheless, our analysis is limited by its retrospective design, the fact that it is based on single center data and the variety of applied (neo)adjuvant treatment measures. Regarding postoperative Gleason's scores, it has to be considered that the number of low Gleason's grades is presumably confounded by tumour regression due to the preoperatively applied antihormonal treatment.

## 5. Conclusions

Although RP might be inadequate as solitary therapeutic approach for high-risk prostate cancer in a subset of patients, the procedure allows surgical control with good quality of life and satisfying functional outcome. Accurate pathologic information and improved patient selection for individual adjuvant treatment is possible. Even in individuals presenting with elevated PSA ≥ 40 ng/mL, RP offers not only long-term disease control in general but also a curative approach in at least 16% of patients with organ-confined disease.

## Figures and Tables

**Table 1:** Preoperative patient characteristics (IQR: interquartile range).

*n* =	56
Age, mean (IQR)	66.81 y (61.2–70.1)
PSA, median (IQR)	54.2 ng/mL (46–79.1)
Followup, median (IQR)	84.83 months (37.57–109.43)
Gleason's score (biopsy)	
≤6	25 (44%)
7	20 (36%)
8–10	11 (20%)
Clinical stage	
cT1	10 (18%)
cT2	9 (16%)
cT3/4	37 (66%)

**Table 2:** Postoperative patient characteristics.

Pathologic Gleason's score	
≤6	17 (30%)
7	26 (46%)
8–10	13 (24%)
Pathologic stage	
pT2	9 (16%)
pT3	20 (36%)
pT4	27 (48%)
Pathologic nodal status	
N0	29 (54%)
N+	25 (46%)
Surgical margin status	
Negative	30 (54%)
Positive	26 (46%)
Hormonal therapy	
Adjuvant	21 (38%)
Neoadjuvant	17 (30%)
None	18 (33%)
Adjuvant RT	
Yes	28 (50%)
No	28 (50%)
Quality-of-life assessment (SF-12): general state of health	
Excellent	6%
Very good	25%
Good	50%
Fair	6%
Poor	13%
Continence assessment (pad usage)	
0-1	58%
1	29%
2 or more	13%
